# Effect of dietary medicinal plants on physiological responses of broiler chickens challenged with *Campylobacter jejuni*

**DOI:** 10.1002/vms3.70028

**Published:** 2024-09-11

**Authors:** Zaynab Nazari, Hassan Shirzadi, Kamran Taherpour, Enayat Rahmatnejad, Ali Khatibjoo

**Affiliations:** ^1^ Department of Animal Science Faculty of Agriculture Ilam University Ilam Iran; ^2^ Department of Animal Science Faculty of Agriculture and Natural Resources Persian Gulf University Bushehr Iran

**Keywords:** blood biochemistry, digestibility, immune response, microbiology, morphology

## Abstract

**Background:**

Phytogenic additives would be helpful to alleviate the adverse effect of *Campylobacter jejuni* on the performance and physiological responses of broiler chickens.

**Objective:**

This experiment was carried out to investigate the effects of *Echinacea purpurea* (**EP**) and *Thymbra spicata* (**TS**) on the performance, nutrient digestibility, serum biochemistry, intestinal morphology, intestinal microbiota and immune responses of broilers challenged with *C. jejuni* from 0 to 42 days of age.

**Methods:**

A total of 240 male broiler chickens were divided into 6 groups and fed various diets: a control diet; the control diet supplemented with EP at 0.25% (EP25) or 0.50% (EP50); the control diet supplemented with TS at 0.25% (TS25) and 0.50% (TS50); or the control diet containing erythromycin at 55 ppm. Chicks were orally challenged with *C. jejuni* on Days 21 and 23 of age.

**Results:**

EP and TS25 diets enhanced European production efficiency factor, feed conversion ratio and digestibility of dry matter and organic matter. TS25 increased duodenal villous height (VH) and surface area on Day 42 of age. EP25 diet increased ileal VH compared to control and erythromycin diets. Diets containing certain EP25 and TS increased the *Bifidobacterium* population and decreased *C. jejuni* population on Day 39 of age. EP50 and TS50 diets increased antibody titration against Newcastle disease virus.

**Conclusions:**

In conclusion, EP and TS dietary supplementation improved performance, microflora, intestinal morphology and immune responses in *C. jejuni*‐challenged broilers.

## Graphical abstract

• This study was carried out to evaluate the effect of *Echinacea purpurea* (**EP**) and *Thymbra spicata* (**TS**) on the broilers challenged with *Campylobacter jejuni*.

• *Campylobacter jejuni* challenge negatively affected performance, nutrient digestibility, intestinal epithelium, gut microbial population and immune response of broiler chickens.

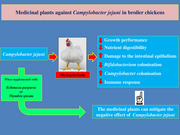

## 1 INTRODUCTION

*Campylobacter* spp., specifically, *Campylobacter jejuni*, colonizes and resides in the gastrointestinal tract (GIT) of poultry, and as such, infected poultry products are considered among the most important causes of foodborne bacterial diseases that afflict human beings both in developed and developing countries (Connerton et al., 2018).

*Campylobacter* can easily colonize and reside in poultry GIT and suppress chickens’ immune responses of chickens (Achen et al., 1998). The pathogens may also cause diarrhoea and increase digesta passage rate (Asuming‐Bediako et al., 2019; Humphrey et al., 2014), shortening digestion and nutrient utilization (Zhang & Adeola, 2016). Diarrhoea also causes wet litter, which can damage poultry health attributable to ammonia gas emission and disorders related to feet and legs because of standing on unwanted litter (Humphrey et al., 2014). The aforementioned pathological outcomes diminish the growth of broiler chickens. Hence, the poultry industry suffers a significant financial loss when *Campylobacter* colonization occurs.

*C. jejuni* and *Campylobacter coli*, as two species at fault for most human diseases, are quite widespread in poultry production containing 80% of the flocks bearing the bacteria. As a result, the degree of bacterial transference to poultry meat increases (Connerton et al., 2018). Consumption of *Campylobacter*‐infected chicken carcass other than fever and diarrhoea can make consumers vulnerable to many diseases, like inflammatory bowel disease, reactive arthritis and Guillain–Barré syndrome (Vandeputte et al., 2019). For instance, most strains of *C. jejuni* contain lipooligosaccharides that structurally mimic human gangliosides. Antibodies against these lipooligosaccharide structures can trigger an autoimmune response which results in Guillain–Barré syndrome (Nemelka et al., 2009).

Broilers just get colonized by *Campylobacter* after 2–3 weeks, which may be because of the protection received from the maternal IgY antibodies (Vandeputte et al., 2019). Since the passage of 2 weeks, the concentration of the antibody blood against *Campylobacter* is decreased significantly, accompanied by a rise in the colonization susceptibility of chickens (Vandeputte et al., 2019). When a bird is infected, the pathogen spreads the infection roughly over the entire flock in 1 week (Vandeputte et al., 2019). Nevertheless, biosecurity may not be a very effective countermeasure to minimize the number of *Campylobacter*‐contaminated poultry due to a large number of pathogens in faeces, leaking intestinal content during the slaughter (Rosenquist et al., 2006) and drug‐resistant strains of *Campylobacter* which develop as a result of the abuse of antimicrobials in poultry (Moore et al., 2006).

Consequently, several alternatives have been devised and extensively studied to decrease the amount of *C. jejuni* in poultry via nonantibiotic approaches (e.g. vaccination, organic acids and probiotics). Furthermore, regarding organic broiler production, medicinal plants have attracted much attention as natural alternatives widely used to control enteric infections in poultry (Attia et al., 2017; Kheiri et al., 2018; Nosrati et al., 2017). Furthermore, most plants containing chemicals with antimicrobial, antiviral, anthelmintic and anti‐oxidative properties are recognized to endocrine and immune systems (Dahiya et al., 2006).

Notwithstanding reports that suggest medicinal plants as possible alternatives to antibiotics effective against *Campylobacter*, the potential of very few of them has been investigated. Inconsistent results regarding substituting phytogenics encourage researchers to conduct studies to understand their role in poultry diets better.

*Echinacea purpurea* (EP) and *Thymbra spicata* (TS) are plants with antimicrobial and antioxidant properties. It has been reported that the main components of EP were total phenols (47.33%), caffeic acid derivatives (30.22%) and cichoric acid (21.76%), respectively (Tsai, Chiou, et al., 2012). Among these phytochemicals, caffeic acid derivatives, especially cichoric acid, strongly show bioactive functions, including antibacterial, antiviral, antioxidant and immunostimulant activities (Tsai, Chiou, et al., 2012; Tsai, Chiu, et al., 2012). Kirkan et al. (2019) reported that carvacrol (63.23%), *γ*‐terpinene (18.94%) and *p*‐cymene (8.31%) were the major components of TS, respectively. Carvacrol, as the major active component (Han & Parker, 2017), has strongly shown antimicrobial and antioxidant, anti‐inflammatory properties, immune‐enhancing activity, inhibition of lipid peroxidation and ability to stimulate digestion (Hashemipour et al., 2013).

Recently, some changes have occurred in chicken microbiota in reaction to *Campylobacter* colonization (Connerton et al., 2018). The hypothesis for the present study was that the colonization of *C. jejuni* in the GIT could disturb the gut microbial balance and cause dysbiosis, which has detrimental effects on the immune responses of broilers and, subsequently, their growth performance. Therefore, the present study attempted to examine the impact of various concentrations of EP or TS, as alternatives to antibiotics, on growth performance, immune responses, intestine morphology, microflora population and particularly their capability to mitigate *Campylobacter* colonization in *C. jejuni*‐challenged broilers.

## 2 MATERIALS AND METHODS

### 2.1 Dietary treatments

Experimental diets were formulated to meet or exceed Ross 308 (Ross, 2014) nutrient recommendations. The control diet (without additives) was formulated based on a corn–soybean meal. Other diets were supplemented with EP at concentrations of 0.25% or 0.50% (EP25 and EP50, respectively), TS at concentrations of 0.25% or 0.50% (TS25 and TS50, respectively) and erythromycin (55 ppm) ranging from 0 to 10 days of age as ‘starter’, 11 to 24 days of age as ‘grower’ and 25 to 42 days of age as ‘finisher’ (Table 1). All broiler chickens were orally challenged with 4 × 10^11^ colony‐forming unit (cfu)/mL *C. jejuni* (RTCC 1097) at 21 and 23 days of age (1 mL per bird in each inoculation). The birds had free access to feed (mash) and water. Chromium oxide (Cr_2_O_3_), an inert marker, was added to all diets (from 38 to 42 days of age) at a concentration of 3 g/kg to determine nutrients digestibility.

**TABLE 1:** Ingredients and nutrient contents of experimental diets at any stage of broiler chickens’ growth.

	Starter	Grower	Finisher
	0–10 days	11–24 days	25–42 days
Ingredient (g/kg)			
Corn	562.0	582.8	632.0
Soybean meal	373.4	346.2	293.2
Sand^a^	5.0	5.0	5.0
Soybean oil	16.6	27.8	33.9
Dicalcium phosphate	18.3	16.3	14.6
Limestone	8.7	7.8	7.4
Sodium chloride	3.0	3.0	3.0
Sodium bicarbonate	1.0	1.0	1.0
l‐Lysine‐HCl	2.3	1.5	1.6
dl‐Methionine	3.4	2.8	2.6
l‐Threonine	1.3	0.8	0.7
Vitamin and mineral premix^b^	5.0	5.0	5.0
Nutrient composition (g/kg, unless otherwise stated)			
Metabolizable energy (kcal/kg)	2900	3000	3100
Crude protein	220	208	189
Calcium	9.3	8.4	7.8
Available phosphorus	4.6	4.2	3.8
Sodium	1.6	1.6	1.6
Lysine	12.4	11.1	10.0
Methionine	6.3	5.6	5.2
Methionine + cysteine	9.2	8.4	7.8
Threonine	8.3	7.5	6.7

^a^ The sand was replaced (wt/wt) by *Echinacea purpurea*, *Thymbra spicata* or erythromycin to form the corresponding experimental diets.

^b^ Supplied as retinyl acetate, 9000 IU; cholecalciferol, 2000 IU; dl‐α‐tocopheryl acetate, 12.5 IU; menadione sodium bisulfite, 1.76 mg; choline chloride, 320 mg; nicotinic acid, 28 mg; calcium d‐pantothenate, 6.4 mg; riboflavin, 3.2 mg; pyridoxine, 1.97 mg; thiamine, 1.2 mg; folic acid, 0.38 mg; biotin, 0.12 mg; cyanocobalamin, 0.01 mg; FeSO_4_·7H_2_O, 80 mg; MnSO_4_·H_2_O, 60 mg; ZnO, 51.74 mg; CuSO_4_·5H2O, 8 mg; iodized NaCl, 0.8 mg; Na_2_SeO_3_, 0.2 mg/kg of diet.

### 2.2 Broiler chickens and experimental design

Two hundred and forty‐day‐old male broiler chicks (Ross 308) were obtained from a commercial hatchery (Parham Joojeh, Rasht, Gilan, Iran). On the first day of age, chicks were weighed (35 g on average), sorted in terms of weight and assigned to pens in an environmentally controlled room. The birds were randomly assigned to treatments using a completely randomized design. The room temperature was held constantly at 32°C in the first 3 days of the trial and gradually decreased by 3°C per week until it reached 20°C. Each of the six experimental diets had five replicates, each containing eight chicks.

### 2.3 Campylobacter challenge

Isolated *C. jejuni* (RTCC 1097) was employed for the experimental challenge (Razi Vaccine and Serum Research Institute, Karaj, Iran). The bacterium was cultured onto *Campylobacter* Selective Agar (Cat No. 1.02249, Merck) and incubated at 42°C in microaerophilic conditions (5% O_2_, 10% CO_2_ and 85% N_2_, produced by the Anoxomat System, Mart Microbiology) in 48 h. Following the incubation, most *C. jejuni* strains produced grey, moist, flat and spreading colonies on the agar. Then, it came to the harvesting of bacteria, where they were cultured onto trypticase soy broth (Cat No. 1224.00) and incubated in microaerophilic conditions. After 5 days, the bacterial colonies were harvested and diluted in phosphate‐buffered saline to the specific viable concentration (4 × 10^11^ cfu/mL). After that, the MacFarlane tubes were used to estimate the inoculum concentration. The inoculum was kept in the refrigerator (4°C) before being gavaged to broilers (inoculation in crop). On Days 21 and 23 of age, the oral challenging of broilers was carried out with a 1.0 mL dose of the inoculum.

### 2.4 Sample collection

On Day 42 of age, a random selection of broiler chickens (three chickens/pen) was made. Then, a blood sample from the left wing vein was collected to determine blood biochemistry, liver enzyme activities and immune response parameters. After blood collection, they were weighed individually, and after being euthanized, their lymphoid organs (thymus, spleen and bursa of Fabricius) were removed and weighed. Subsequently, 3–4 cm of duodenal, jejunal and ileal middle sections were cut and fixed in 10% neutral buffered formalin to measure intestinal morphology. Finally, on Day 42 of age, fresh excreta samples were collected from each pen to determine microflora counts and apparent nutrient digestibility.

### 2.5 Growth performance

Feed intake (FI) was obtained as the difference between feed quantity offered and refused over 0–10, 11–24 and 25–42 days of age. Moreover, body weight gain (BWG) was obtained during the abovementioned periods. Accordingly, the time required to calculate the European production efficiency factor (EPEF) and feed conversion ratio (FCR) was ripe.

### 2.6 Apparent digestibility of nutrients

All diets and fresh excreta samples from each pen were freeze‐dried and ground via a 0.5‐mm screen and were stored at −20°C until further analyses of the nutrient contents were conducted. Then, based on the method described by AOAC (1990), ash, dry matter (DM) and crude protein (CP) were specified, and ash was subtracted from DM to obtain organic matter (OM). Chromium was determined based on the method proposed by Nguyen et al. (2018). Briefly, the samples were ashed in a muffle furnace (Iso‐temp D3714, Fisher Scientific) at 600°C for 24 h in porcelain crucibles and then digested in citric acid. The mixture was heated slowly for 40 min and incubated in HClO_4_ for 3 h at 140°C. Next, an atomic absorption spectrometer was used to read the absorbance at 405 nm (AA 670; Shimadzu). In addition, the standard digestibility equation was employed to calculate the coefficients of apparent nutrient digestibilities (Nguyen et al., 2018).

### 2.7 Serum biochemistry and liver enzyme activities

Serum samples were collected (centrifuged at 2000 × *g* for 10 min at room temperature). Total protein, triglyceride (TG), cholesterol (CHOL), high‐density lipoprotein (HDL), low‐density lipoprotein (LDL), alanine aminotransferase (ALT) and aspartate aminotransferase (AST) activities were assessed using specific kits (Pars Azmoon) and a spectrophotometer (BT1500).

### 2.8 Small intestinal morphology

The small intestine segments (duodenal, jejunal and ileal tissues) were fixed in paraformaldehyde, dehydrated with ethanol and xylene and then embedded in paraffin. The tissues were sectioned into 5 µm thick pieces using a LEICA RM 2145 microtome (LEICA, JENA). Finally, slides were stained using haematoxylin and eosin. The villous width (VW), villous height (VH) and crypt depth (CD) were also assessed, and then, the villous surface area (VSA) and VH to CD ratio (villous index, VI) were calculated (Sakamoto et al., 2000). The mean value of 10 adjacent, vertically oriented villous‐crypt units per section was noted to conduct the required analyses.

### 2.9 Excreta microbial shedding

On Days 21 and 39 of age, the collected fresh excreta samples (approximately 15 min after excreted) from each pen were transferred on ice to the microbial laboratory before and after the challenge, respectively. One gram of each sample was diluted by phosphate‐buffered saline, and serial dilutions (10^−3^–10^−7^) were subsequently prepared. Finally, five 20 µL volumes of each dilution were plated on each media. The de Man, Rogosa and Sharpe agar (Cat No. QB‐65‐5262, Quelab), *Bifidobacterium* selective agar (Cat No. B1143, Biomark), *Campylobacter* selective agar (Cat No. 1.02249, Merck), brilliance phenol red green agar (Cat No. QB‐65‐5236, Quelab) and plate count agar (Cat No. QB‐65‐5240, Quelab) media were used for isolation of *Lactobacillus*, *Bifidobacterium*, *C. jejuni*, *Salmonella* and total aerobic bacteria, respectively. *C. jejuni* was counted following the microaerophilic incubation at 42°C for 24 h. The number of other bacteria was calculated after incubation in an aerobic chamber at 37°C for 24 h. Afterwards, logarithmic (log_10_) transformation was utilized on the microbial cfu.

### 2.10 Immunological assay

Two sheep red blood cell (SRBC; 0.5 mL, 7%) antigen injections were performed intramuscularly in the left breast muscle to evaluate humoral system responses at 28 and 35 days of age. After selecting the injected birds from all replicate pens, it was time to take the blood samples via wing vein at Day 42 of age. Subsequently, serum was separated by centrifugation of coagulated blood at 3000 rpm for 7 min. Total immunoglobulin (IgT), and IgTs M, and G (IgM and IgG, respectively) were specified by haemagglutination inhibition (HI). Furthermore, HI was used to measure antibody titration against the avian influenza virus (AIV) and Newcastle disease virus (NDV). EDTA‐mixed blood samples were examined for heterophils, eosinophils, lymphocyte and monocyte counts (Biasato et al., 2017), and the heterophil/lymphocyte ratio (HLR) was calculated. The weights of the thymus, spleen and bursa of Fabricius were taken and stated as a body weight percentage.

### 2.11 Statistical analysis

The SAS software package (SAS Inst. Inc., 2004) was employed for all statistical analyses. The pen was utilized as the experimental unit, and data analysis was conducted using a completely randomized design with the GLM procedure. Tukey's multiple comparison tests were run to compare the significant differences (*p *< 0.05).

## 3 RESULTS

### 3.1 Growth performance

Table 2 shows that there was no effect of dietary treatments on BWG and FI during all experimental periods, and FCR and EPEF of broiler chickens from Days 0 to 10 and 11 to 24 of age (*p *> 0.05). However, birds fed dietary treatments containing certain concentrations of medicinal plants had decreased FCR compared with birds fed control diet from Days 25 to 42 and 0 to 42 of age (*p < *0.05), except the one containing TS50. Furthermore, birds fed diet supplemented with erythromycin had decreased FCR from Days 25 to 42 of age (*p *< 0.05). Birds fed diets supplemented with EP50 and TS25 had increased EPEF from 25 to 42 days; those fed diets containing EP50, EP25, TS25 and erythromycin had increased EPEF from 0 to 42 days of age when compared with the control (*p *< 0.05). There was no significant difference in mortality rate (*p > *0.05; data not shown).

**TABLE 2:** Effects of dietary treatments on growth performance of broiler chickens challenged with *Campylobacter jejuni*.

	Treatment^a^							
Item	Control	EP25	EP50	TS25	TS50	Eryth	SEM	*p*‐value
Starter (0–10 days)								
Body weight gain (g/bird)	221	220	236	242	237	238	7	0.16
Feed intake (g/bird)	257	265	263	263	259	269	6	0.78
Feed conversion ratio	1.16	1.20	1.11	1.09	1.10	1.13	0.03	0.04
EPEF	190	184	206	215	210	204	10	0.22
Grower (11–24 days)								
Body weight gain (g/bird)	701	737	681	742	691	736	34	0.68
Feed intake (g/bird)	1177	1184	1167	1175	1187	1233	35	0.80
Feed conversion ratio	1.71	1.61	1.72	1.59	1.72	1.69	0.09	0.81
EPEF	304	329	284	335	289	319	28	0.72
Finisher (25–42 days)								
Body weight gain (g/bird)	1262	1352	1423	1455	1347	1391	58	0.28
Feed intake (g/bird)	3257	3011	3048	3051	3126	3133	70	0.21
Feed conversion ratio	2.58^a^	2.23^b^	2.15^b^	2.10^b^	2.32^ab^	2.28^b^	0.07	<0.01
EPEF	273^b^	338^ab^	371^a^	378^a^	312^ab^	345^ab^	25	0.05
Overall (0–42 days)								
Body weight gain (g/bird)	2184	2309	2340	2439	2275	2364	67	0.20
Feed intake (g/bird)	4690	4459	4477	4488	4572	4635	98	0.48
Feed conversion ratio	2.15^a^	1.93^b^	1.92^b^	1.85^b^	2.01^ab^	1.96^ab^	0.04	<0.01
EPEF	242^c^	285^ab^	281^ab^	307^a^	252^bc^	277^ab^	11	<0.01

*Note*: Means within a row sharing a common superscript (^a,b,c^) are not different (*p* > 0.05).

Abbreviation: EPEF, European production efficiency factor.

^a^ EP25; diet containing *Echinacea purpurea* at levels of 0.25%, EP50; diet containing *Echinacea purpurea* at levels of 0.50%, TS25; diet containing *Thymbra spicata* at levels of 0.25%, TS50; diet containing *Thymbra spicata* at levels of 0.50%, Eryth; erythromycin (55 ppm).

### 3.2 Apparent digestibility of nutrients

The effects of different treatments on the apparent nutrient digestibility of broilers are shown in Table 3. No significant difference was found in faecal DM content (*p *> 0.05). The TS25 group led to an increase in CP digestibility when compared with the control diet (*p *< 0.05). Dietary treatments increased ash digestibility when compared with the control diet (*p *< 0.05). Offering diets containing EP25, EP50 or TS25 increased DM digestibility compared to the control and TS50 diets (*p *< 0.05). Furthermore, the OM digestibility increased in broilers fed diets containing EP and TS25 (*p < *0.05).

**TABLE 3:** Effects of dietary treatments on broiler chickens’ faecal digestibility challenged with *Campylobacter jejuni*.

Item	Treatment^a^						SEM	*p*‐value
	Control	EP25	EP50	TS25	TS50	Eryth		
Faecal digestibility								
Faecal DM	16.8	17.2	16.8	16.0	17.9	16.4	0.8	0.67
CP digestibility	73.3^b^	75.9^ab^	76.4^ab^	78.1^a^	75.3^ab^	74.7^ab^	0.9	0.04
Ash digestibility	56.6^c^	64.5^a^	62.6^ab^	60.0^b^	62.5^ab^	61.2^ab^	0.7	<0.01
DM digestibility	76.4^b^	80.7^a^	80.5^a^	81.0^a^	76.9^b^	79.6^ab^	1.0	0.02
OM digestibility	80.4^b^	83.7^a^	83.2^a^	83.1^a^	82.3^ab^	82.4^ab^	0.5	<0.01

*Note*: Means within a row sharing a common superscript (^a,b,c^) are not different (*p* > 0.05).

Abbreviations: CP, crude protein; DM, dry matter; OM, organic matter.

^a^ EP25; diet containing *Echinacea purpurea* at levels of 0.25%, EP50; diet containing *Echinacea purpurea* at levels of 0.50%, TS25; diet containing *Thymbra spicata* at levels of 0.25%, TS50; diet containing *Thymbra spicata* at levels of 0.50%, Eryth; erythromycin (55 ppm).

### 3.3 Serum biochemistry and liver enzyme activities

The effects of different treatments on broilers’ serum metabolite profiles and liver enzyme activities are presented in Table 4. On Day 42 of age, no significant differences were found in total protein, CHOL, HDL, LDL, AST and ALT (*p > *0.05). However, diets containing EP25 and TS25 resulted in lower and higher TG, respectively, compared to the other diets (*p < *0.05).

**TABLE 4:** Effects of dietary treatments on serum biochemistry, liver enzyme activities of broiler chickens challenged with *Campylobacter jejuni*.

Item	Treatment^a^						SEM	*p*‐value
	Control	EP25	EP50	TS25	TS50	Eryth		
Total protein (g/dL)	8.38	9.28	9.68	9.35	7.23	8.15	0.76	0.25
TG (mg/dL)	59.5^b^	33.0^c^	60.0^b^	87.8^a^	47.8^bc^	50.0^bc^	5.9	<0.01
CHOL (mg/dL)	85.3	73.3	90.5	81.8	82.0	60.0	7.7	0.13
HDL (mg/dL)	34.3	30.3	37.3	29.5	33.3	25.5	3.4	0.25
LDL (mg/dL)	39.1	36.4	41.3	34.7	39.2	24.5	4.4	0.16
AST (U/L)	133	107	108	122	100	111	12	0.39
ALT (U/L)	2.75	1.50	3.00	2.00	4.50	1.50	0.76	0.09

*Note*: Means within a row sharing a common superscript (^a,b,c^) are not different (*p* > 0.05).

Abbreviations: ALT, alanine aminotransferase; AST, aspartate aminotransferase; CHOL, cholesterol; HDL, high‐density lipoprotein; LDL, low‐density lipoprotein; TG, triglyceride.

^a^ EP25; diet containing *Echinacea purpurea* at levels of 0.25%, EP50; diet containing *Echinacea purpurea* at levels of 0.50%, TS25; diet containing *Thymbra spicata* at levels of 0.25%, TS50; diet containing *Thymbra spicata* at levels of 0.50%, Eryth; erythromycin (55 ppm).

### 3.4 Small intestinal morphology

Table 5 shows data on the small intestinal morphology of broiler chickens. On Day 42 of age, the diet containing TS25 caused a significant increase in VH, and diets containing TS25 and erythromycin significantly increased VSA of the duodenum compared with the control diet (*p *< 0.05). The VH of ileum was increased by diets containing EP25 compared with the control and erythromycin‐incorporated diets (*p *< 0.05). In this regard, no statistically significant difference was observed between the excremental diets in duodenal VW, CD and VI; all morphological characteristics of the jejunum; as well as ileal VW, CD, VI and VSA (*p *> 0.05).

**TABLE 5:** Effects of dietary treatments on small intestinal morphology of broiler chickens challenged with *Campylobacter jejuni*.

Item	Treatment^a^						SEM	*p*‐value
	Control	EP25	EP50	TS25	TS50	Eryth		
Duodenum								
Villus height (µm)	1465^b^	1576^ab^	1521^ab^	1809^a^	1526^ab^	1594^ab^	66	0.02
Villus width (µm)	161	184	177	193	169	216	13	0.09
Crypt depth (µm)	216	221	209	201	207	182	17	0.67
Villus height:crypt depth	6.90	7.90	7.37	9.16	7.39	8.99	0.65	0.10
Villus surface area (mm^2^)	0.735^b^	0.912^ab^	0.850^ab^	1.108^a^	0.802^ab^	1.081^a^	0.078	0.01
Jejunum								
Villus height (µm)	994	1000	1052	1007	977	1167	50	0.11
Villus width (µm)	154	153	107	156	156	124	14	0.06
Crypt depth (µm)	161	146	171	138	173	180	11	0.08
Villus height:crypt depth	6.22	6.91	6.19	7.51	5.75	6.62	0.43	0.09
Villus surface area (mm^2^)	0.483	0.465	0.354	0.501	0.479	0.453	0.047	0.33
Ileum								
Villus height (µm)	821^b^	968^a^	840^ab^	930^ab^	839^ab^	793^b^	33	<0.01
Villus width (µm)	205	194	178	179	158	199	21	<0.01
Crypt depth (µm)	170	172	161	147	141	169	12	0.38
Villus height:crypt depth	5.04	5.75	5.32	6.52	6.02	4.96	0.50	0.22
Villus surface area (mm^2^)	0.529	0.602	0.473	0.519	0.417	0.494	0.051	0.24

*Note*: Means within a row sharing a common superscript (^a,b^) are not different (*p* > 0.05).

^a^ EP25; diet containing *Echinacea purpurea* at levels of 0.25%, EP50; diet containing *Echinacea purpurea* at levels of 0.50%, TS25; diet containing *Thymbra spicata* at levels of 0.25%, TS50; diet containing *Thymbra spicata* at levels of 0.50%, Eryth; erythromycin (55 ppm).

### 3.5 Excreta microbial shedding

The effects of different dietary treatments on the excreta microbial shedding of broilers are presented in Table 6. Before the birds were challenged with *C. jejuni* on Day 21 of age, no statistically significant difference was observed between birds fed different diets (*p > *0.05; unpublished data). After the birds were challenged with *C. jejuni*, on Day 39 of age, no significant differences were observed in the populations of *Lactobacillus*, *Salmonella* and total aerobic bacteria (*p > *0.05). Offering diets containing additives significantly increased the *Bifidobacterium* population compared with the control diet (*p* < 0.05). However, no difference was found between the diets containing EP50 or erythromycin and the control diet (*p > *0.05). All diets used except EP50 led to a significant decrease in *C. jejuni* population compared with the control diet (*p *< 0.05); no significant difference was observed between the EP50 and the control diet (*p > *0.05).

**TABLE 6:** Effects of dietary treatments on broiler chickens’ excreta microbial shedding challenged with *Campylobacter jejuni*.

Item (log_10_)	Treatment^a^						SEM	*p*‐value
	Control	EP25	EP50	TS25	TS50	Eryth		
Faecal microbiology								
*Lactobacillus*	10.30	9.97	10.60	10.00	10.40	9.72	0.30	0.40
*Bifidobacterium*	7.94^b^	9.14^a^	8.32^ab^	8.86^a^	9.13^a^	8.76^ab^	0.19	<0.01
*Campylobacter jejuni*	11.30^a^	9.88^bc^	10.70^ab^	10.10^bc^	9.36^c^	9.77b^c^	0.22	<0.01
*Salmonella*	8.30	7.92	8.76	8.43	9.30	8.34	0.46	0.42
Total aerobic bacteria	12.60	12.00	11.70	11.90	11.80	11.80	0.30	0.34

*Note*: Means within a row sharing a common superscript (^a,b,c^) are not different (*p* > 0.05).

^a^ EP25; diet containing *Echinacea purpurea* at levels of 0.25%, EP50; diet containing *Echinacea purpurea* at levels of 0.50%, TS25; diet containing *Thymbra spicata* at levels of 0.25%, TS50; diet containing *Thymbra spicata* at levels of 0.50%, Eryth; erythromycin (55 ppm).

### 3.6 Immunity responses

Table 7 shows data on immunity responses and lymphoid organ weights of broilers. Offering different diets had not any significant effect on antibody titre against SRBC (IgT, IgG and IgM), antibody titre against AIV, eosinophil and monocyte counts, or relative lymphoid organ weights (*p > *0.05). Additionally, diet containing TS50 increased antibody titre against NDV (*p < *0.05). Diets incorporated with TS led to a significant decrease in lymphocyte counts and a significant increase in heterophil counts and HLR (*p < *0.05).

**TABLE 7:** Effects of dietary treatments on immunity responses and lymphoid organ weights of broiler chickens challenged with *Campylobacter jejuni*.

Item	Treatment^a^						SEM	*p*‐value
	Control	EP25	EP50	TS25	TS50	Eryth		
Antibody titre against SRBC (log_2_)								
IgT	5.00	5.75	5.00	5.25	5.00	4.75	0.53	0.83
IgG	3.50	3.75	3.75	3.25	3.50	3.25	0.39	0.89
IgM	1.50	2.00	1.25	2.00	1.50	1.50	0.33	0.52
Antibody titre against Newcastle disease and avian influenza viruses (log_2_)								
NDV	1.25^c^	2.50^abc^	3.50^ab^	1.75^bc^	4.25^a^	2.75^abc^	0.39	<0.01
AIV	2.75	3.00	3.50	3.25	3.25	4.00	0.43	0.44
White blood cells								
Lymphocyte (%)	69.3^a^	67.5^ab^	66.8^ab^	60.3^bc^	55.3^c^	66.3^ab^	1.7	<0.01
Heterophil (%)	27.8^c^	30.0^bc^	30.3^bc^	38.0^ab^	39.3^a^	31.0^abc^	2.0	<0.01
Eosinophil (%)	1.50	2.00	2.00	1.25	3.50	2.00	0.18	0.26
Monocyte (%)	1.50	0.50	1.00	0.50	2.00	0.75	0.12	0.04
HLR	0.403^c^	0.450^bc^	0.458^bc^	0.635^ab^	0.720^a^	0.468^bc^	0.048	<0.01
Relative lymphoid organ weights (%)								
Thymus	0.103	0.108	0.088	0.108	0.093	0.095	0.017	0.94
Spleen	0.113	0.098	0.108	0.113	0.108	0.110	0.013	0.96
Bursa of Fabricius	0.200	0.248	0.203	0.228	0.220	0.228	0.028	0.85

*Note*: Means within a row sharing a common superscript (^a,b,c^) are not different (*p* > 0.05).

Abbreviations: AIV, avian influenza virus; HLR, heterophil/lymphocyte ratio; IgG, immunoglobulin G; IgM, immunoglobulin M; IgT, total immunoglobulin; NDV, Newcastle disease virus, SRBC, sheep red blood cell.

^a^ EP25; diet containing *Echinacea purpurea* at levels of 0.25%, EP50; diet containing *Echinacea purpurea* at levels of 0.50%, TS25; diet containing *Thymbra spicata* at levels of 0.25%, TS50; diet containing *Thymbra spicata* at levels of 0.50%, Eryth; erythromycin (55 ppm).

## 4 DISCUSSION

It is proven that phytogenic components with antimicrobial and antioxidant properties can effectively improve poultry performance and maintain intestinal health (Kheiri et al., 2018). Although some researchers have reported positive effects of EP on broilers’ performance, others have found no effect of EP on broilers (Nosrati et al., 2017) and pigs (Maass et al., 2005), as well as the effect of TS on broilers (Attia et al., 2017). These findings are consistent with the results of the present study. Moreover, according to the present study, the addition of EP (Lee et al., 2012; Rahimi et al., 2011) and TS (Rahimi et al., 2011) to broilers diets decreases FCR. This improvement is attributed to enhanced digestive enzyme secretions (Rahimi et al., 2011) and the antibacterial and antifungal properties of EP and TS (Souri et al., 2015). The property reduces harmful bacteria while promoting beneficial bacteria, thereby improving feed digestibility (Ragaa et al., 2016) and increasing efficiency. In general, the present study revealed that incorporating medicinal plants and erythromycin into diets does not affect BWG and FI, and it could partially improve the FCR.

Diet components can extensively affect the GIT microflora of poultry. Overall, all treatments in the present study reduced the number of *C. jejuni* and increased the number of *Bifidobacterium* in broilers infected by *C. jejuni* compared with the control diet. The results from the present study agree with those of Ebrahimi et al. (2016), who showed that EP significantly decreases cecal *C*. *jejuni* colonization but has no effect on the number of colonies in the liver tissue. They attributed this to the feed additives, which only had a limited effect on the GIT contents. Witkowska and Sowińska (2013) reported that thyme possesses antibacterial effect, especially against *Escherichia coli* and *Staphylococcus aureus*. Furthermore, it has been noted that TS has strong antibacterial and antioxidant effects due to its high levels of phenolic and flavonoid components (Bayan et al., 2017).

Thymol and carvacrol are the main effective components of TS, playing a key role in its antibacterial effect (Thanissery & Smith, 2014). These compounds can decrease S*almonella enteritidis* and *C*. *jejuni* (Venkitanarayanan et al., 2013), as well as *E. coli* and *Salmonella typhimurium* (Toghyani et al., 2010) in GIT of broilers. Additionally, when medicinal plants suppress the colonization of pathogenic bacteria, beneficial bacteria can proliferate (Zhen et al., 2018). This observation is supported by the increased *Bifidobacterium* population noted in the present study. Therefore, dietary supplementation with medicinal plants can beneficially alter the cell membrane integrity (Hernandez et al., 2004) and counteract *C. jejuni*, thereby increasing the *Bifidobacterium* population.

Medicinal plant extracts improve the digestibility and performance of broilers (Witkowska & Sowińska, 2013). Medicinal plant extracts can ensure optimum levels of pathogenic bacteria colonization as well as nonpathogenic (Hernandez et al., 2004), which can provide more nutrients for the host and improve digestibility (Hernandez et al., 2004). In addition, the EP contains putative pharmacological components (e.g. phenolic and alkamides), which stimulate enzyme secretion and lead to better feed utilization (Nasir & Grashorn, 2010). In contrast to the present study, Cross et al. (2011) found that thymol does not significantly affect broilers’ digestibility and performance. The differences in basal diet and (level of) experimental materials can explain these variations, at least partially. Accordingly, improvements in nutrient digestibility observed in broilers fed EP and TS are likely to result from improved intestinal microflora (Table 6) and intestinal morphology (Table 5).

As reported by researchers, medicinal plants promote the intestinal morphology of monogastric animals. Antimicrobial agents can indirectly influence VH and CD by decreasing intestinal pathogenic load (Zhen et al., 2018). An improvement in VH attributed to the usage of thymol has been claimed in prior research (Ragaa et al., 2016), consistent with the present study. A considerable improvement in VH caused by dietary treatments can lead to an increase in VSA of the duodenum. This can increase the absorption of nutrients, and it may partially explain the observed improvement in FCR. However, it is not clear regarding the other intestinal segments. A crypt can be considered a villous factory, and a large crypt indicates a fast tissue turnover and high demand for the generation of new tissues (Xu et al., 2003). Any additional tissue turnover demands a relatively higher quantity of nutrients needed for maintenance and, as such, will reduce animal efficiency (Xu et al., 2003). Thus, unaffected CD in the presence of dietary treatments indicates a normal intestinal proliferative.

Normal crypts can indicate usual demands for the proliferation of cells and renewal of tissues, which leads to better feed efficiency. Therefore, the VI is considered a desired criterion for estimating the digestive capacity of small intestine. Including mint powder in the quail diet improves ileal VI (Mehri et al., 2015). Similar results were obtained by the increase of VH and VI in the intestines of *Bacillus coagulans*‐fed broilers challenged with *S. enteritidis* compared to the control group (Zhen et al., 2018). The authors have explained that this was related to the increased intestinal tissue turnover due to increased beneficial bacteria (Mehri et al., 2015; Zhen et al., 2018), which matches with the present study; a finding confirmed by the measured microflora population (Table 6). Broilers fed the dietary treatments showed unchanged VI, which implies that the treatments did not reduce the digestive capacity of nutrients in the small intestine. Therefore, increased duodenal and ileal VH have to result from an improvement in intestinal microflora population in the presence of EP and TS (Table 6), which may reduce toxic metabolites by pathogenic bacteria.

Metabolite concentrations and activities of liver enzymes in the serum are the criteria for addressing the metabolic, nutrition and health status of animals. They can be used to assay the consequences of medical plants on the metabolism, immunity and physiological responses of broilers (Zhu et al., 2014). In contrast, it is impossible to understand the detailed in vivo mechanism(s) of lipid metabolism affected by medicinal plant sources.

The beneficial effects of EP powder on blood lipid profiles have been reported as decreased CHOL and increased HDL in layers (Jahanian et al., 2017) and as decreased TG and CHOL in broilers (Nosrati et al., 2017). It is, however, clear that the proliferation of some anaerobic organisms (e.g. *Lactobacillus*, *Bifidobacteria* and *Enterococci*) can lead to the deconjugation of bile salts (Saeed et al., 2015). As TG is one of the main components of micelles, the decrease in TG caused by EP can be due to the deconjugation of bile salts and the proliferation of some bacteria, as indicated by the increase in *Bifidobacterium* population observed in the present study (Table 6). On the other hand, thymol can decrease TG, increase HDL in Japanese quails (Kheiri et al., 2018) and increase HDL in broilers (Toghyani et al., 2010). It has been reported that this is because of the roles of thymol and carvacrol in the reduction of liver 3‐hydroxy‐3‐methylglutaryl coenzyme A, which is considered an important enzyme in the synthesis of CHOLs (Kheiri et al., 2018) and lipolysis processes (Toghyani et al., 2010). This can explain the findings of the current study.

The dietary treatments in the present study did not influence liver enzyme activities; similar results have also been obtained in another research project in which medicinal plants had positive effects on the liver health of pigs, whereas their serum liver enzyme activities remained unchanged (Saeed et al., 2015). Furthermore, thymol does not change blood metabolites such as CHOL, HDL and LDL (Souri et al., 2015) and ALT and AST (Ragaa et al., 2016), which may indicate that thymol does not affect liver and kidney stability.

Increased total protein levels, decreased AST caused by dietary thymol in broilers (Attia et al., 2017) and increased HDL (Zhu et al., 2014) have also been reported, which is inconsistent with the present results. Variations in medicinal plant delivery, applied medicinal plant concentrations, differences in bioactive components and basal diet may explain these discrepancies.

Studies have generally discovered that EP (Lee et al., 2012) and thymol (Rahimi et al., 2011) have antibiotic and immunostimulant properties. Research findings have argued that EP can strengthen the immune system in poultry (Skopinska‐Rozewska et al., 2010). It has also been proven that dietary EP root extract significantly decreases the injuries in the intestines of poultry that have experienced contamination on the part of the coccidia. It also improves the birds’ health compared to the control (Allen, 2003). Immunostimulant properties of *Echinacea* spp. have been referred to in many studies, and this characteristic is likely to activate the lymphoid organs to a larger degree. Moreover, it has been identified that EP administration may raise the angiogenic activity of spleen cells (Skopinska‐Rozewska et al., 2010).

Allen (2003) has found that EP can improve immune responses by stimulating natural killer cells and macrophages in broilers challenged with live oocyst vaccine and may be effective against the incidence of coccidiosis. Böhmer et al. (2009) obtained similar results in the case of improved antibody titres against SRBC in broilers using EP to enhance the immune system. In contrast, antibody titre against SRBC and lymphoid organ weights remained unchanged in the present study. Nevertheless, Ghazvinian et al. (2017) have demonstrated that using 200 mg/kg of *Zataria multiflora* in broiler diet positively affects SRBC response; in other words, this concentration can stimulate the immune system. However, unaffected SRBC has also been reported after using thymol (Souri et al., 2015) which is in line with the results of the current study. The particular function of lymphoid organs makes them susceptible to oxidative injury. Therefore, the antioxidant activity of dietary treatments is likely to protect them against oxidative injuries and can make it possible for antibody‐producing organs to carry out their functions highly efficiently.

As noted previously, *Echinacea* spp. positively impacts the whole immune system, which can be traced to nonspecific and specific immune responses. This result of the present research is in harmony with Böhmer et al. (2009) finding, where it was revealed that the ethanolic extract of *Echinacea* significantly increases the frequency of lymphocytes and total leukocytes among hens and pigs. Ebrahimi et al. (2016) reported that EP increases HLR compared to the control diet. Increases in heterophil, HLR, and decreases in lymphocytes have been reported due to using thymol (Souri et al., 2015). This is in contrast to the present study in which the usage of EP decreased heterophil and HLR while increasing lymphocytes. The main reasons may be the dosage of the medicinal plants used in this study and the applied *C. jejuni* challenge load.

Rahimi et al. (2011) have shown that EP has the biggest impact on the immune system with no effect on the red and white blood cells in pigs (Maass et al., 2005), consistent with this research. It is reported that EP has potential effects against AIV (Najafzadeh et al., 2011). Increased antibody titration against NDV due to the usage of *Cichorium intybus* has been reported, which is in line with the results of the current study (Saeed et al., 2015). It has been attributed to the antimicrobial properties of *C. intybus*, a finding that could further explain the results obtained from this study. Hashemipour et al. (2013) reported increased cellular and humoral immune response caused by thymol and carvacrol, the main components of thyme, used in broilers' diet, which is consistent with the present study. Some unaffected immunity parameters such as blood monocytes, heterophil and eosinophil (Attia et al., 2017), lymphatic organelles weights (Hashemipour et al., 2013; Souri et al., 2015) and antibody titration against SRBC (Toghyani et al., 2010) have also been argued.

## 5 CONCLUSIONS

The results of this study indicate that supplementing the diet of broiler chickens with EP and TS at a concentration of 0.25% can improve growth performance. Additionally, EP and TS supplementation can enhance intestinal morphology and certain immunological parameters, such as antibody titration against NDV in *C. jejuni*‐challenged broilers.

## AUTHOR CONTRIBUTIONS

**Zaynab Nazari**: Methodology; data curation; project administration. **Hassan Shirzadi**: Supervision; conceptualization; investigation; methodology; data curation; funding acquisition; Writing—original draft. **Kamran Taherpour**: Conceptualization; investigation; methodology; data curation. **Enayat Rahmatnejad**: Investigation; validation; data analysis; writing—review and editing. **Ali Khatibjoo**: Investigation; methodology; validation; data analysis.

## CONFLICT OF INTEREST STATEMENT

The authors declare no conflicts of interest.

## ETHICS STATEMENT

All experimental protocols adhered to the guidelines, which were approved by the Animal Ethics Committee of Ilam University (Ilam, Iran) and were in accordance with the EU standards for the protection of animals and/or feed legislation. The ethical approval number based on the submitted MSc student thesis proposal is 1397/02, Anim Sci Dep.

## Data Availability

The data that support the findings of this study are available on the request of readers via the email address provided.
